# Modifiable Individual Risks of Perioperative Blood Transfusions and Acute Postoperative Complications in Total Hip and Knee Arthroplasty

**DOI:** 10.3390/jpm11111223

**Published:** 2021-11-18

**Authors:** Axel Jakuscheit, Nina Schaefer, Johannes Roedig, Martin Luedemann, Sebastian Philipp von Hertzberg-Boelch, Manuel Weissenberger, Karsten Schmidt, Boris Michael Holzapfel, Maximilian Rudert

**Affiliations:** 1 Department of Orthopaedic Surgery, University of Wuerzburg, Koenig-Ludwig-Haus, Brettreichstr. 11, 97070 Wuerzburg, Germany; nina-schaefer@web.de (N.S.); joroedig@googlemail.com (J.R.); m-luedemann.klh@uni-wuerzburg.de (M.L.); s-boelch.klh@uni-wuerzburg.de (S.P.v.H.-B.); m-weissenberger.klh@uni-wuerzburg.de (M.W.); m-rudert.klh@uni-wuerzburg.de (M.R.); 2 Department of Trauma, Hand, Plastic and Reconstructive Surgery, University Hospital Würzburg, Josef-Schneider-Str. 2, 97080 Wuerzburg, Germany; schmidt_k@ukw.de; 3 Department of Orthopedics and Trauma Surgery, Musculoskeletal University Center Munich, Marchionistr. 15, 81377 Munich, Germany; boris.holzapfel@med.uni-muenchen.de

**Keywords:** patient blood management, total joint arthroplasty, haemoglobin, perioperative management

## Abstract

Background: The primary aim of this study was to identify modifiable patient-related predictors of blood transfusions and perioperative complications in total hip and knee arthroplasty. Individual predictor-adjusted risks can be used to define preoperative treatment thresholds. Methods: We performed this retrospective monocentric study in orthopaedic patients who underwent primary total knee or hip arthroplasty. Multivariate logistic regression models were used to assess the predictive value of patient-related characteristics. Predictor-adjusted individual risks of blood transfusions and the occurrence of any perioperative adverse event were calculated for potentially modifiable risk factors. Results: 3754 patients were included in this study. The overall blood transfusion and complication rates were 4.8% and 6.4%, respectively. Haemoglobin concentration (Hb, *p* < 0.001), low body mass index (BMI, *p* < 0.001) and estimated glomerular filtration rate (eGFR, *p* = 0.004) were the strongest potentially modifiable predictors of a blood transfusion. EGFR (*p* = 0.001) was the strongest potentially modifiable predictor of a complication. Predictor-adjusted risks of blood transfusions and acute postoperative complications were calculated for Hb and eGFR. Hb = 12.5 g/dL, BMI = 17.6 kg/m^2^, and eGFR = 54 min/mL were associated, respectively, with a 10% risk of a blood transfusion, eGFR = 59 mL/min was associated with a 10% risk of a complication. Conclusion: The individual risks for blood transfusions and acute postoperative complications are strongly increased in patients with a low preoperative Hb, low BMI or low eGFR. We recommend aiming at a preoperative Hb ≥ 13g/dL, an eGFR ≥ 60 mL/min and to avoid a low BMI. Future studies must show if a preoperative increase of eGFR and BMI is feasible and truly beneficial.

## 1. Introduction

The probabilities of blood transfusions and perioperative complications in total joint arthroplasty (TJA) are highly influenced by patient-related risk factors such as age and comorbidities [1,2]. To lower the patients’ individual risks by a target-orientated preoperative treatment, it is essential to identify potentially modifiable risk factors.

A frequently reported modifiable risk factor is a low preoperative haemoglobin (Hb) concentration, which is not only associated with a higher rate of blood transfusions but also renal, cardiac and wound-related complications [3]. Since a low Hb concentration can often be successfully treated, an anaemia screening and the treatment of preoperative anaemia has become a foremost aim in the run-up for TJA [4]. However, it remains unclear which minimum Hb concentration should be aimed at to effectively reduce the rate of blood transfusions and perioperative complications. In particular, previous studies are controversial if female sex is an independent risk factor [5,6] and if different Hb thresholds should be used in male and female patients [7]. While a too high threshold may lead to a higher transfusion rate, a too low threshold may result in a treatment of non-diseased asymptomatic patients who are by definition not ill, which is not only a relevant cost factor but also a medicolegal dilemma.

Therefore, we performed this study to investigate the predictive value of the preoperative Hb concentration and other patient-related risk-factors for blood transfusions and acute postoperative complications. On the basis of this data, we aimed to define useful target values of a preoperative treatment.

## 2. Methods

We performed this monocentric retrospective study after approval of the local ethics committee (Ethikkommission der Medizinischen Fakultät der Universität Würzburg, application number AZ-2018071001) and completed registration at the German register for clinical studies (Deutsches Register Klinischer Studien, registration number DRKS00015219).

### 2.1. Data Collection

We collected the data of all patients that underwent elective total hip and bicondylar knee arthroplasty between January 2016 and December 2018 in a single orthopaedic university hospital.

Demographic, anamnestic and clinical data were collected retrospectively from the hospital’s information technology system (ORBIS, Agfa Healthcare GmbH, Bonn, Germany). Data about the use of allogeneic and autologous blood transfusions were crosschecked using hard copy records. Of the demographic and anamnestic data, we recorded the patient’s age, sex, height and weight as well as the preoperative medication, daily consumption of alcohol or nicotine and comorbidities. Of the preoperative clinical data, we recorded the American Society of Anaesthesiologists (ASA) status and the lab values of the last blood sample before undergoing surgery, including c-reactive protein (mg/dL), haemoglobin concentration (g/dL), haematocrite (%), mean corpuscular volume (MCV) (fl), platelet count (10^3^/µL), creatinine (µmol/L), estimated glomerular filtration rate (eGFR) (mL/min) Quick (%) and partial thromboplastin time (PTT) (sec).

Of the intraoperative data, we collected the type of anaesthesia, the type of surgery, the use of tranexamic acid, duration of surgery, the use of drains and the use of an autologous re-transfusion system (cell saver). 

### 2.2. Outcome Measures

The administration of at least one allogeneic red blood cell (RBC) transfusion was the primary outcome of this investigation. According to the hospital’s guideline, an Hb concentration <6 g/dL was an unconditional trigger for RBC transfusions. A Hb concentration <8 g/dL was a conditional trigger regarding the patient’s individual resources and clinical symptoms. Blood transfusions in patients with a Hb concentration >8 g/dL were well-founded exceptions.

The secondary outcome measure was the occurrence of an adverse event during the patient’s stay in hospital that required an unexpected change of treatment. 

### 2.3. Statistical Analysis

Statistical analysis was performed on deidentified data using SPSS Statistics 26 (IBM inc., Armonk, NY, USA).

To investigate the predictive power of the preoperative characteristics, we calculated logistic regression models for both outcome measures. To include only the strongest predictors, we modified the inclusion and exclusion criteria for the regression models. To predict an allogeneic RBC transfusion, a variable was included in the model when its inclusion improved the model fit with a significance of *p* ≤ 0.01. Variables were removed from the model, when adding of further variables reduced the significance of the variable dependent improvement to *p* ≥ 0.05.

To predict a complication, a variable was included in the model when its inclusion improved the model fit with a significance of *p* ≤ 0.05. Variables were removed from the model when adding of further variables reduced the significance of the variable dependent improvement to *p* ≥ 0.1.

In case the logistic regression models revealed a potentially modifiable risk factor for the outcome measures, we calculated a monovariate logistic regression model for the respective risk factor to receive a continuous estimation of the predictor-adjusted risks. 

## 3. Results

We analyzed the data of 2109 female and 1645 male patients who underwent total joint arthroplasty. The mean age (±standard deviation, SD) was 66.5 (±11.1) years, the mean body mass index (±SD) was 29.5(±5.8) kg/m^2^. The mean ASA score (IQR) was 2 (2–3). The most common comorbidities were high blood pressure (63.9%), cardiovascular disease (22.2%), hypothyreosis (17.6%) and pulmonary disease (17.3%). The mean (±SD) preoperative haemoglobin (Hb) concentration was 14.1 (±1.4) g/dL and the mean (±SD) estimated glomerular filtration rate (eGFR) was 84.5 (±21.2) mL/min. Further comorbidities and preoperative lab values are shown in Table 1. 

A total of 1806 patients underwent primary bicondylar arthroplasty via the medial parapatellar approach and 1948 patients underwent primary total hip arthroplasty via the direct anterior approach according to Hueter [8]. Mean duration of surgery was 86.6 (±23.2) minutes, 1013 (27%) patients received intravenous tranexamic acid, 461 (12.3%) received local tranexamic acid and in 625 patients (16.6%) an autologous blood transfusion was used (Table 1).

Allogeneic red blood cell (RBC) transfusions were applied in 179 (4.8%) patients. Adverse events during the patient’s stay in hospital were recorded in 239 (6.4%) patients.

In the multivariate logistic regression model for an allogeneic RBC transfusion, the preoperative Hb concentration (*p* < 0.001), cardiovascular disease (*p* < 0.001), low body mass index (*p* < 0.001), diabetes (*p* = 0.001), ASA score (*p* = 0.001), haemophilia (*p* = 0.002), thrombocytes (*p* = 0.003), estimated glomerular filtration rate (eGFR, *p* = 0.004) and age (*p* = 0.027) were the strongest predictors. In the multivariate logistic regression model for a perioperative complication the ASA status (*p* < 0.001), age (*p* < 0.001), eGFR (*p* = 0.001) and Quick (0.002) were the strongest predictors (Table 2).

**Figure 1:**
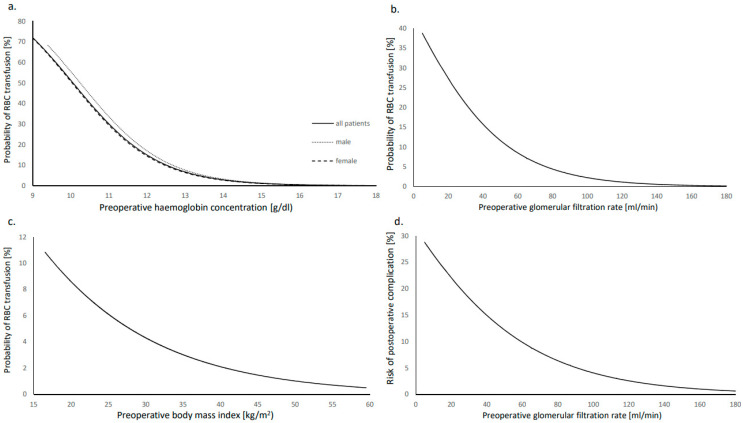
Predicted probabilities of perioperative allogeneic red blood cell (RBC) transfusion with respect to the preoperative haemoglobin concentration (**a**), preoperative estimated glomerular filtration rate (eGFR) (**b**) and preoperative body mass index (**c**). Predicted risks of the occurrence of an acute postoperative complication with respect to the preoperative eGFR (**d**).

Predictor-adjusted risks of blood transfusions were calculated for preoperative Hb concentration, BMI and eGFR (Figure 1a–c). Predictor-adjusted risks of acute postoperative complications were calculated for eGFR (Figure 1). The individual transfusion probability exceeded 10% in patients with a Hb concentration < 12.6 g/dL, BMI < 17.9 kg/m^2^ or eGFR < 55 mL/min.

The individual risk for an acute postoperative complication exceeded 10% in patients with an eGFR < 60 mL/min. 

To estimate the risks of blood transfusions in a cohort of high-risk patients with a low Hb concentration (Hb < 13 g/dL), low BMI (<22 kg/m^2^) [9] or a low eGFR (eGFR < 60 mL/min), we calculated the respective odds ratios (OR) with 95% confidence interval (CI) (Table 3). To estimate the risks of acute postoperative complications in high-risk patients with a low eGFR (eGFR < 60 mL/min), we calculated the respective OR with 95% CI For allogeneic blood transfusions, the OR (95% CI) was 8.02 (5.87–10.96) in patients with Hb < 13 g/dL, 4.83 (3.48–6.68) in patients with eGFR < 60 mL/min, and 2.51 (1.39–4.56) in patients with BMI < 22 kg/m^2^, respectively.

For acute postoperative complications, the OR (95% CI) was 2.75 (2–3.78) in patients with eGFR < 60 mL/min. Within the recorded kinds of complications, the OR (95% CI) was significantly increased for cardiac complications (2.45 (1.27–4.7)), falls (3.37 (1.18–9.61)) and renal complications (8.84 (5.16–15.16)) (Table 3).

## 4. Discussion

This study was performed to investigate potentially modifiable patient-related risk factors for blood transfusions and acute postoperative complications in total joint arthroplasty. Our results show that numerous patient-related characteristics are potential risk factors for blood transfusion (Table 2). Some of the identified characteristics are non or hardly modifiable such as the patient’s age and comorbidities, which were in our study as cardiovascular diseases, haemophilia, low thrombocytes and ASA status. Provided that all patients receive adequate treatment of their comorbidities, these characteristics are non-modifiable unless by choosing an earlier time-point of surgery in the patient’s life. An earlier surgery might not only reduce the patient’s age but also age-related diseases [10], contributing to a lower transfusion rate. This is in line with the results of previous studies, which showed that younger and healthier patients have a lower risk for blood transfusions [6,11].

In our study, we focused on potentially modifiable predictors of blood transfusions such as a low body mass index (BMI), a low estimated glomerular filtration rate (eGFR) and a low haemoglobin (Hb) concentration. Our results confirm the results of a previous study that a high body mass index is a protective factor for blood transfusions in major surgery [12]. Since a high body weight is associated with a high blood volume [13] but not with a high blood loss [14], patients with a high BMI undergo a relatively low blood loss and are therefore less prone to blood transfusions [12]. However, considering the negative effects of a high BMI [15] we recommend avoiding not only malnutrition but also obesity if a long-term preparation for TJA is possible.

In contrast, a low Hb concentration is a highly predictive factor of a blood transfusion that can also be addressed by a short-term preoperative treatment [16] using iron supplementation [17,18] and erythropoietin [19]. However, it remains unclear which Hb concentration is an optimum threshold for an efficient preoperative treatment, not least because of sparse data about the effect of iron supplementation in non-anaemic patients [20]. Different thresholds have already been proposed by previous studies [6,7]. As the surgical approaches and blood sparing techniques might differ between medical centres, it was our aim to find useful thresholds for our standard techniques in primary hip and knee arthroplasty. An important finding was that the patient’s sex and type of TJA were not significant predictors of blood transfusions. Therefore, we calculated the Hb-adjusted probability of blood transfusions regardless of the patient’s sex and type of TJA (Figure 1). To illustrate the difference in the Hb-adjusted risks of male and female patients, we added the separated curves to Figure 1. 

Although in our study the overall rate of transfusions was under 5%, our results showed that many patients undergo a much higher individual probability of a blood transfusion. For instance, if the Hb concentration was 12.5 g/dL or less, the individual transfusion risk exceeded 10% (Figure 1). In our opinion, it is not appropriate to use the WHO criteria of anaemia [21] to decide whether a patient should receive a preoperative treatment or not. In line with previous results [22], we recommend aiming at a Hb concentration of at least 13 g/dL in the run-up for primary TJA in men and women.

Another finding of our study showed that the preoperative estimated glomerular filtration rate (eGFR) was a predictive factor of blood transfusions as well, which has been reported before [6,23]. Possibly, a low eGFR is not an independent risk factor but is only associated with a risk factor that we did not account for. However, we considered numerous patient-related characteristics (Table 1) to calculate our logistic regression model. Therefore, impaired renal function is likely a true risk factor for blood transfusions. We calculated an eGFR-adjusted individual probability of blood transfusions that showed a value of at least 60 mL/min is associated with an individual transfusion probability under 10%. 

On the basis of the Hb concentration (13 g/dL), BMI (22 kg/m^2^) and eGFR (60 mL/min) thresholds, we created “high-risk” groups to illustrate the impact on the transfusion rate (Table 3). The results show that patients with a Hb under 13 g/dL have an eight-fold higher risk of a transfusion and patients with an GFR under 60 mL/min a 5-fold and patients with a BMI under 22 kg/m^2^ an almost 3-fold higher risk of a blood transfusion. Future studies must show if a target orientated preoperative treatment in such patients results in a higher Hb concentration, BMI and eGFR to lower the transfusion rate. 

Our results further showed that a low eGFR is not only associated with a higher rate of blood transfusions but also with a higher risk of acute postoperative complications, mainly acute renal insufficiency but also cardiac complications and a higher risk of falling. According to the individual eGFR-adjusted risks, patients with an eGFR < 60 mL/min had an individual risk for a complication of more than 10% while the overall risk in our population was 6.4%. In patients with an eGFR under 40 mL/min, the risk of a complication even exceeded 20%. This is in line with sparse previous results that reported renal insufficiency as a risk factor for complications [23]. Probably our rather sensitive outcome considering also mild adverse events resulted in a clearer identification of a low eGFR as a risk factor. We recommend screening every patient for low eGFR in the run-up for TJA. At least in patients with an eGFR < 60 mL/min, a diagnostic work-up should be initiated.

An increase of the preoperative eGFR might result in a decrease of transfusions and perioperative complications. Probably, at least a small portion of patients will benefit from such a ‘’patient kidney management“, as some types of renal insufficiency might be modifiable by a higher intake of water [24,25] or an improved treatment of cardiovascular and renal diseases [26,27].

Such management to improve and protect kidney function should not only call for preoperative but also intra- and postoperative measures such as controlling renal perfusion [27], avoiding nephrotoxic medication and recognizing acute renal dysfunction as soon as possible. In future, some medication might contribute to kidney protection, but evidence is missing [28].

Interestingly, the preoperative Hb concentration was not a significant predictive factor for acute complications in our multivariate logistic regression model although preoperative anaemia is a frequently reported risk factor for complications [3,29]. This finding suggests that in some patients not the preoperative Hb concentration itself but associated characteristics such as higher age and ASA status are the underlying cause for acute postoperative complications [30].

### Limitations

Several limitations of this retrospective study must be addressed. Due to its retrospective nature, we do not know if the potentially modifiable risk factors are truly modifiable. Second, we do not know if a treatment of these risk factors, even if it changes the risk factor’s value, truly lowers the risk of blood transfusions and complications. However, at least for a low Hb concentration, previous studies have already shown that a successful treatment results in a lower transfusion rate [17].

Another limitation of our study is that the uses of tranexamic acid, drains and cell savers were left at the discretion of the responsible anaesthesiologist and surgeon. This results in a high variability of the individual treatment and therefore reduces the accuracy of predictor-adjusted individual risks. To estimate a patient’s individual risk for a transfusion or complication, many more than the potentially modifiable risk factors investigated here must be considered. Moreover, to address the high variability of the investigated characteristics, multi-centre studies with a high number of patients are indispensable.

Follow-up studies are crucial for investigating the modifiability of Hb, BMI and eGFR and the usefulness of the thresholds recommended here. These thresholds will have to be updated regularly based on their effect on changes in rates of transfusions and complications. In addition, further development in preoperative treatment as well as in surgical and anaesthesiologic techniques will have to be regarded.

## 5. Conclusions

Our results confirm that a low Hb concentration is a main risk factor for blood transfusion. Men and women with a preoperative Hb concentration <13 g/dL undergo an 8-fold higher risk of blood transfusions, and the individual risk for a blood transfusion exceeds 10% in patients with a preoperative Hb of less than 12.6%. We recommend aiming at a minimum preoperative Hb concentration of 13 g/dL. 

The preoperative estimated glomerular filtration rate (eGFR) is also a significant risk factor for blood transfusions but also for acute postoperative complications. Patients with an eGFR under 60 mL/min had a five-fold higher risk of transfusions and three-fold higher risk of complications. Future studies must show if it is possible to increase the eGFR in the run-up for total joint arthroplasty and if the transfusion and complication rates can be further reduced.

The third modifiable risk factor for blood transfusions was a low BMI. Therefore, malnutrition should also be addressed during “prehabilitation” for total joint arthroplasty.

## Figures and Tables

**Table 1:** Clinical characteristics.

Preoperative Characteristics	
Age, mean ± SD (years)	66.5 ± 11.1
Sex, *n* (female/male)	2109/1645
Body Mass Index, mean ± SD (kg/m^2^)	29.5 ± 5.8
ASA score, median (IQR)	2 (2–3)
*Medical history, n (%)*	
Cardiovascular disease	834 (22.2)
Pulmonary disease	649 (17.3)
Renal disease	246 (6.6)
Diabetes	534 (14.2)
High blood pressure	2399 (63.9)
Hypothyreosis	660 (17.6)
Smoker	450 (12)
Depression	179 (4.8)
Rheumatic disease	70 (1.9)
Thrombophilia	235 (6.3)
Haemophilia	20 (0.5)
Long-term aspirin	672 (17.9)
Long-term anticoagulation	358 (9.5)
*Blood parameters*	
Haemoglobin, ±SD (g/dL)	14.1 ± 1.4
Haematokrit, ±SD (%)	42.4 ± 3.9
Thrombocytes, ±SD (10^3^/µL)	242.9 ± 61.8
Serum creatinine, ±SD (µmol/L)	77.8 ± 27.7
eGFR, ±SD (mL/min)	84.5 ± 21.2
C-reactive protein (mg/dL)	0.6 ± 0.9
PTT (s)	34.2 ± 4.6
Quick (%)	100.4 ± 13.0
Intraoperative characteristics	
Type of surgery, *n* (hip arthroplasty/knee arthroplasty)	1948/1806
Use of i.v. tranexamic acid, *n* (%)	1013 (27.0)
Use of local tranexamic acid, *n* (%)	461 (12.3)
Type of anaesthesia (general/neuraxial)	2247/1507
Duration of surgery, mean ± SD (min)	86.6 ± 23.2
Autologous RBC transfusion, *n* (%)	625 (16.6)
Postoperative characteristics	
Allogeneic RBC transfusion, *n* (%)	179 (4.8)
Perioperative complications, *n* (%)	239 (6.4)
Wound site complications (SSI/PJI)	20 (0.5)
Periprosthetic fracture	6 (0.2)
Prosthesis malposition/dislocation	5 (0.1)
Need for Intensive Care	17 (0.5)
Postoperative delirium	30 (0.8)
Decubitus	20 (0.5)
Cardiac complications	52 (1.4)
Pneumonia	10 (0.3)
Fall	17 (0.5)
Hepatic complication	5 (0.1)
Neurological complications	22 (0.5)
Renal complications	55 (1.5)
Gastrointestinal complications	27 (0.7)
DVT/LE	3 (0.1)

SD—standard deviation, ASA—American Association of Anesthesiologist, —glomerular filtration rate, PTT—partial thromboplastin time, i.v.—intravenous, RBC—red blood cell, OR—odds ratio, CI—confidence interval, sig—significance, RBC—red blood cell, DVT—deep vein thrombosis, LE—lung artery embolism.

**Table 2:** Logistic regression models to predict allogeneic RBC transfusion (a) and perioperative complications (b).

Preoperative Variables	B	S.E.	Exp (B)	*p*
(a)				
Constant	8.182	1.484	3576.322	<0.001
Haemoglobin	−0.779	0.069	0.459	<0.001
Cardiovascular disease	0.897	0.192	2.453	<0.001
Body Mass Index	−0.068	0.018	0.934	<0.001
Diabetes	−0.941	0.282	0.390	0.001
ASA score	0.581	0.181	1.788	0.001
Haemophilia	2.088	0.690	8.067	0.002
Thrombocytes	−0.004	0.001	0.996	0.003
eGFR	−0.012	0.004	0.988	0.004
Age	0.021	0.009	1.021	0.027
Nagelkerke R Square	0.320			
(b)				
Constant	−4.652	0.949	0.010	<0.001
ASA score	0.545	0.131	1.724	<0.001
Age	0.044	0.008	1.045	<0.001
eGFR	−0.012	0.003	0.988	0.001
Quick	−0.015	0.005	0.985	0.002
Nagelkerke R Square	0.096			

B—regression coefficient; S.E.—standard error of regression coefficient; Exp (B)—exponentiation of coefficient, *p*—significance of each predictive variable in the model.

**Table 3:** Rates and risks of allogeneic red blood cell transfusions and complications in potentially modifiable high-risk patients.

	Preop. eGFR < 60 mL/min *n* = 416 (11.1%)	OR (95%CI)	Preop. Hb < 13 g/dl *n* = 666 (17.7%)	OR (95% CI)	Preop. BMI < 22 kg/m^2^ *n* = 198 (5.3%)	OR (95% CI)
Outcome Measures						
RBC transfusion, n (%)	62 (14.9)	4.82 (3.48–6.68)	197 (16.1)	8.02 (5.87–10.96)	19 (9.6)	2.51 (1.39–4.56)
Complications, n (%)	57 (13.7)	2.75 (2.00–3.78)				
Wound site complications	2 (0.5)	0.89 (0.21–3.85)				
Periprosthetic fracture	2 (0.5)	4.02 (0.74–22.04)				
Prosthesis malposition/migration	1 (0.2)	2.01 (0.22–18.0)				
Need for Intensive Care	4 (1.0)	2.48 (0.81–7.65)				
Postoperative delirium	4 (1.0)	1.24 (0.43–3.56)				
Decubitus	1 (0.2)	0.42 (0.06–3.15)				
Cardiac complications	12 (2.9)	2.45 (1.27–4.7)				
Pneumonia	1 (0.2)	0.89 (0.11–7.05)				
Fall	5 (1.2)	3.37 (1.18–9.61)				
Hepatic complication	1 (0.2)	2.0 (0.22–18.0)				
Neurological complications	3 (0.7)	1.27 (0.37–4.3)				
Renal complications	28 (6.7)	8.84 (5.16–15.16)				
Gastrointestinal complications	6 (1.4)	2.31 (0.93–5.76)				
DVT/LE	1 (0.2)	4.02 (0.36–44.4)				

Preop.—preoperative, Hb—haemoglobin, eGFR—estimated glomerular filtration rate, OR—odds ratio, CI—confidence interval, sig—significance, RBC—red blood cell, DVT—deep vein thrombosis, LE—lung artery embolism.

## Data Availability

The data presented in this study are available on request from the corresponding author. The data are not publicly available in accordance with the approval of the local ethics committee.
